# Long noncoding RNA highly upregulated in liver cancer promotes the progression of hepatocellular carcinoma and attenuates the chemosensitivity of oxaliplatin by regulating miR‐383‐5p/vesicle‐associated membrane protein‐2 axis

**DOI:** 10.1002/prp2.815

**Published:** 2021-07-05

**Authors:** Peng Li, Yuwei Li, Lieting Ma

**Affiliations:** ^1^ Department of Laboratory First Affiliated Hospital of Xi'an Jiaotong University Xi’an PR China; ^2^ Department of Genetic Center Northwest Women’s and Children’s Hospital Xi’an PR China

**Keywords:** hepatocellular carcinoma, HULC, miR‐383‐5p, Oxa chemosensitivity, VAMP2

## Abstract

We aimed to explore the function and underlying mechanism of highly upregulated in liver cancer (HULC; an long noncoding RNAs) in hepatocellular carcinoma (HCC) and chemosensitivity of oxaliplatin (Oxa). The expression of HULC, miR‐383‐5p, and vesicle‐associated membrane protein‐2 (VAMP2) was detected by quantitative real‐time polymerase chain reaction. Western blot assay was applied for measuring the protein expression of cyclinD1, cleaved‐caspase‐3, light Chain 3 I/II, p62, and VAMP2. Cell viability and Oxa IC50 value were determined by Cell Counting Kit‐8 assay. A colony formation assay was conducted to evaluate colony formation ability. Cell apoptosis was assessed by flow cytometry. The interaction between miR‐383‐5p and HULC or VAMP2 was predicted by bioinformatics analysis and verified by dual‐luciferase reporter assay and RNA immunoprecipitation assay. The mice xenograft model was established to investigate the roles of HULC in vivo. HULC and VAMP2 were overexpressed whereas miR‐383‐5p was lowly expressed in HCC tissues. HULC overexpression promoted the progression of HCC cells and inhibited chemosensitivity of Oxa by increasing cell proliferation and protective autophagy and inhibiting apoptosis, whereas HULC silence presented opposite effects. Moreover, miR‐383‐5p was a direct target of HULC and miR‐383‐5p reversed the effects of HULC on the progression of HCC cells and chemosensitivity of Oxa. Besides, HULC acted as a molecular sponge of miR‐383‐5p to regulate VAMP2 expression. HULC promoted the progression of HCC and inhibited Oxa sensitivity by regulating miR‐383‐5p/VAMP2 axis, elucidating a novel regulatory mechanism for chemosensitivity of Oxa and providing a potential lncRNA‐targeted therapy for HCC.

## Graphical abstract

HULC promoted HCC progression and inhibited Oxa sensitivity via regulating miR‐383‐5p/VAMP2 axis.

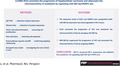

**Abbreviations**
3′UTR: 3′‐untranslated region
CCK‐8: Cell Counting Kit‐8
HCC: hepatocellular carcinoma
HULC: highly upregulated in liver cancer
LC3: light Chain 3
lncRNAs: long noncoding RNAs
miRNAs: microRNAs
NC: negative control
Oxa: oxaliplatin
PI: propidium iodide
qRT‐PCR: quantitative real‐time polymerase chain reaction
RIPA: RNA immunoprecipitation assay
VAMP2: vesicle‐associated membrane protein‐2
V‐FITC: V‐fluorescein isothiocyanate

## 1 INTRODUCTION

Hepatocellular carcinoma (HCC) is one of the most frequent human malignancies in the world and it accounts for 85%–90% of primary liver cancer.^1^, ^2^ Over the past few decades, despite advances in the treatment of HCC, such as operation technique, chemotherapeutic, and radiotherapeutic approaches, the prognosis remains very poor because most of the HCC patients are usually diagnosed at advanced stages.^3^ Autophagy, a conserved catabolic process, is responsible for providing nutrition (ATP, amino acids, etc.) for cell survival by disposing and recycling cellular proteins and damaged/excessive organelles during nutritional deficiency.^4^ Previous studies have demonstrated that multiple chemotherapeutic drugs, such as cisplatin, 5‐fluorouracil, and sorafenib can induce autophagy in HCC cells, decreasing cell apoptosis and chemosensitivity, resulting in the survival of HCC cells.^5^, ^6^ Oxaliplatin (Oxa), a third‐generation platinum anti‐cancer drug, is extensively used in chemotherapeutic regimes to treat advanced HCC.^7^ However, Oxa resistance is one of the most vital barriers for patients with HCC. Hence, it is essential to better understand the molecular mechanisms of HCC and improve the chemosensitivity of Oxa.

Long noncoding RNAs (lncRNAs), one type of endogenous cellular RNAs (>200 nucleotides), lack protein‐coding potential and have pivotal roles in a variety of biological processes.^8^ LncRNAs have been suggested to participate in multiple cellular processes, such as cell differentiation, cell cycle, apoptosis, and autophagy.^9^ Besides, some reports have shown that lncRNAs also could regulate chemosensitivity in cancer cells.^10^, ^11^ LncRNA highly upregulated in liver cancer (HULC), an important lncRNA with a wide variety functions, is specifically overexpressed and serves as an oncogenic lncRNA in human HCC.^12^ Although HULC‐triggered autophagy has been shown to reduce the chemosensitivity of Oxa,^13^ more functions of HULC and its underlying mechanism are still largely unknown in HCC progression and chemosensitivity of Oxa.

A large number of evidence has shown that lncRNAs can function as competing endogenous RNAs (ceRNAs) through sponging microRNAs (miRNAs) to modulate gene expression.^14^, ^15^ MiRNAs are a kind of short noncoding RNAs and modulate gene levels via binding with the 3′‐untranslated region (3′UTR) of target mRNAs, resulting in mRNAs degradation or suppression of their translation.^16^, ^17^ Emerging evidence has revealed that aberrant expression of miRNAs is tightly related to the occurrence and development of HCC as well as chemosensitivity.^18^, ^19^ MiR‐383‐5p has been suggested to play an anti‐cancer role in many tumors, including HCC.^20^ However, the interaction between HULC and miR‐383‐5p and the biological functions of miR‐383‐5p in chemosensitivity of Oxa remain unclear. Vesicle‐associated membrane protein‐2 (VAMP2) has been demonstrated to be expressed at a high level in HCC tissues and cells. In view of these findings, we are interested in whether miR‐383‐5p exerts its effect by regulating VAMP2 expression.

In this research, we determined the abundance of HULC, miR‐383‐5p, and VAMP2 in HCC tissues and HCC cells treated with Oxa. Moreover, the roles of HULC, miR‐383‐5p, and VAMP2 in cell growth, apoptosis, and autophagy were examined in HCC cells treated with Oxa. Besides, we also probed the regulatory network of HULC/miR‐383‐5p/VAMP2 in HCC cells. The aim of this study was to provide a new insight and treatment strategy for Oxa‐based chemotherapeutics.

## 2 MATERIALS AND METHODS

### 2.1 Patient samples

HCC tissues (*n* = 35) and adjacent normal tissues (*n* = 35) were acquired from patients who had undergone surgery at the First Affiliated Hospital of Xi'an Jiaotong University. Before surgery, these patients never received local or systemic treatment. The tissue samples were collected, and promptly frozen in liquid nitrogen, and then cryopreserved at −80°C for subsequent experiments. This research was permitted by the Research Ethics Committee of First Affiliated Hospital of Xi'an Jiaotong University. Informed consent had acquired from each patient.

### 2.2 Cell culture and transfection

Human HCC cells (Hep3B and Huh7) and human embryonic kidney cells (293T) were bought from COBIOER. Cells were grown in RPMI 1640 medium (Invitrogen) containing 10% (v/v) fetal bovine serum (Invitrogen) in a humidified atmosphere with 5% CO_2_ at 37°C. Oxa (Sigma‐Aldrich) was applied to treat Hep3B and Huh7 cells.

Highly upregulated in liver cancer or VAMP2 overexpression vector (HULC or VAMP2) and their negative control (NC; Vector), small interfering RNA against HULC or VAMP2 (si‐HULC or si‐VAMP2) and their NC (si‐NC), miR‐383‐5p mimic and its NC (mimic NC), and miR‐383‐5p inhibitor and its NC (inhibitor NC) were bought from GenePharma. Lipofectamine 3000 reagent (Invitrogen) was utilized to transfect cells.

### 2.3 Quantitative real‐time polymerase chain reaction

Trizol reagent (Invitrogen) WAS applied for isolating total RNA. After that, the RNA samples were reversely transcribed to complementary DNA using the TIANScript RT Kit (Tiangen Biotech). Detection of HULC, VAMP2, and miR‐383‐5p was performed using the SYBR green PCR kit (TaKaRa) on the ABI 7900 system (Applied Biosystems). The 2^−ΔΔ^ *^Ct^* method was employed to calculate the expression of genes. β‐actin and U6 were served as endogenous controls. In this study, primers used for quantitative real‐time polymerase chain reaction (qRT‐PCR) were exhibited as below: HULC, sense: 5′‐ATCTGCAAGCCAGGAAGAGTC‐3′, antisense: 5′‐CTTGCTTGATGCTTTGGTCTGT‐3′; miR‐383‐5p, sense: 5′‐GGGAGATCAGAAGGTGATT‐3′, antisense: 5′‐CAGTGCGTGTCGTGGAGT‐3′; VAMP2, sense: 5′‐CTCCAAACCTCACCAGTAACAGG‐3′, antisense: 5′‐AGCTCCGACAGCTTCTGGTCTC‐3′; β‐actin, sense: 5′‐GAGCTACGAGCTGCCTGAC‐3′, antisense: 5′‐CCTAGAAGCATTTGCGGTGG‐3′; U6, sense: 5′‐CTCGCTTCGGCAGCACATATACT‐3′, antisense: 5′‐ACGCTTCACGAATTTGCGTGTC‐3′.

### 2.4 Western blot assay

Tissue samples and cultured cells were lysed using the RNA immunoprecipitation assay (RIPA) buffer mixed with protease inhibitors (Sigma‐Aldrich), followed by quantitation with the BCA protein assay kit (Applygen). Protein (40 µg per lane) was separated on sulfate‐polyacrylamide gel electrophoresis (SDS‐PAGE), followed by being transferred to nitrocellulose membranes (Beyotime). After blocking with 5% nonfat‐dried milk, membranes were then incubated using the primary antibodies against light Chain 3 (LC3)I/II (1:3000, ab51520; Abcam), p62 (1:1000, ab56416; Abcam), cyclinD1 (1:2000, ab226977; Abcam), cleaved‐caspase‐3 (1:500, ab49822; Abcam), or β‐actin (1:2500, ab8227; Abcam) for 12 h at 4°C. After being washed, the secondary antibody (1:4000, ab205718; Abcam) was used to incubate the membranes for 2 h. Protein bands were visualized by an enhanced chemiluminescent kit (Applygen). Protein expression was normalized to β‐actin and quantified using ImageJ software.

### 2.5 Cell viability assay

Cell Counting Kit‐8 (CCK‐8; Beyotime) was applied for measuring cell viability. In brief, Hep3B and Huh7 cells (5 × 10^3^ per well) were plated in a 96‐well plate. CCK‐8 (10 μl) reagent was added to the wells after treatment, followed by incubation for 3 h. The optical density value at 450 nm was recorded under a microplate reader (Bio‐Rad). Oxa concentration causing 50% inhibition of growth (IC50) was measured using the relative survival curve.

### 2.6 Colony formation assay

Transfected Hep3B and Huh7 cells were plated in 6‐well plates replacing the medium every 3 days. Following incubation for 2 weeks, these cells were fixed using paraformaldehyde (4%) and stained with Giemsa solution (Sigma‐Aldrich). After that, the number of colonies (a colony was defined as >50 cells) was calculated.

### 2.7 Cell apoptosis assay

Cell apoptosis was measured by flow cytometry using Annexin V‐fluorescein isothiocyanate (FITC)/propidium iodide (PI) apoptosis detection kit (Key GEN BioTECH). Briefly, Hep3B and Huh7 cells were collected, re‐suspended in 1×  binding buffer (300 μl) containing Annexin V‐FITC (10 μl) and PI (5 μl) in the darkness for 15 min. Lastly, the cells were subjected to flow cytometry (Partec AG) for detecting the number of apoptotic cells.

### 2.8 Dual‐luciferase reporter assay

The bioinformatics tool miRcode was employed to predict the target gene of HULC, and HULC might interact with miR‐383‐5p. Prediction of miR‐383‐5p binding sites was performed using starbase v3.0, and miR‐383‐5p might bind with the 3′UTR of VAMP2. The HULC or VAMP2 3′UTR fragment containing putative or mutated miR‐383‐5p binding sites was synthesized and inserted into pmirGlO luciferase reporter vector (Promega), generating wild‐type or mutant‐type (HULC‐wt, VAMP2 3′UTR‐wt, HULC‐mut, VAMP2 3′UTR‐mut) luciferase reporter vectors. 293T cells were co‐transfected with one of the reporter vectors and miR‐383‐5p mimic or mimic NC. Following cultivation for 48 h, a dual‐luciferase assay system (Promega) was applied to assess the luciferase activity.

### 2.9 RIP assay

RNA immunoprecipitation assay was performed using the EZ‐Magna RIP Kit (Millipore). First, Hep3B and Huh7 cells were harvested, lysed by complete RIP lysis buffer, followed by incubation with RIP buffer supplemented with magnetic beads conjugated to a human anti‐Ago2 anti‐Ago or anti‐IgG (as control). After that, these samples were incubated using the proteinase K to digest proteins, followed by isolating the immunoprecipitated RNA. The enrichment of miR‐383‐5p or HULC was measured using qRT‐PCR.

### 2.10 Xenograft mice model

Animal experiments were granted by the committee of Animal Research of First Affiliated Hospital of Xi'an Jiaotong University. Vector or oe‐HULC was transfected into Hep3B cells. Afterward, stably transfected cells (5 × 10^6^) were implanted into the BALB/c nude mice (female, 6 weeks old, *n* = 5/group) through subcutaneous injection. Three days later, all mice were treated with Oxa (5 mg/kg) twice a week via tail vein injection for 4 weeks. From the 9th day, tumor volume was monitored by examining the length (*L*) and width (*W*) using the calipers every 3 days and calculated using the following formula: volume = (*L* × *W* ^2^)/2. After 4 weeks, the mice were sacrificed using cervical dislocation, tissue samples were weighed and collected for further studies.

### 2.11 Statistical analysis

The data were presented as mean ± standard deviation and the experiments were repeated at least 3 times in this study. Correlation between miR‐383‐5p and HULC or VAMP2 was analyzed using the Spearman rank correlation. Statistical analyses were performed by GraphPad Prism 6.0 (GraphPad Software Inc.). The Student's *t*‐test (for 2 groups) or a one‐way analysis of variance (ANOVA; for multiple groups) was used to compare values of test and control samples. *p* < .05 was regarded as statistically significant.

## 3 RESULTS

### 3.1 HULC was overexpressed and Oxa‐activated autophagy in HCC

**FIGURE 1:**
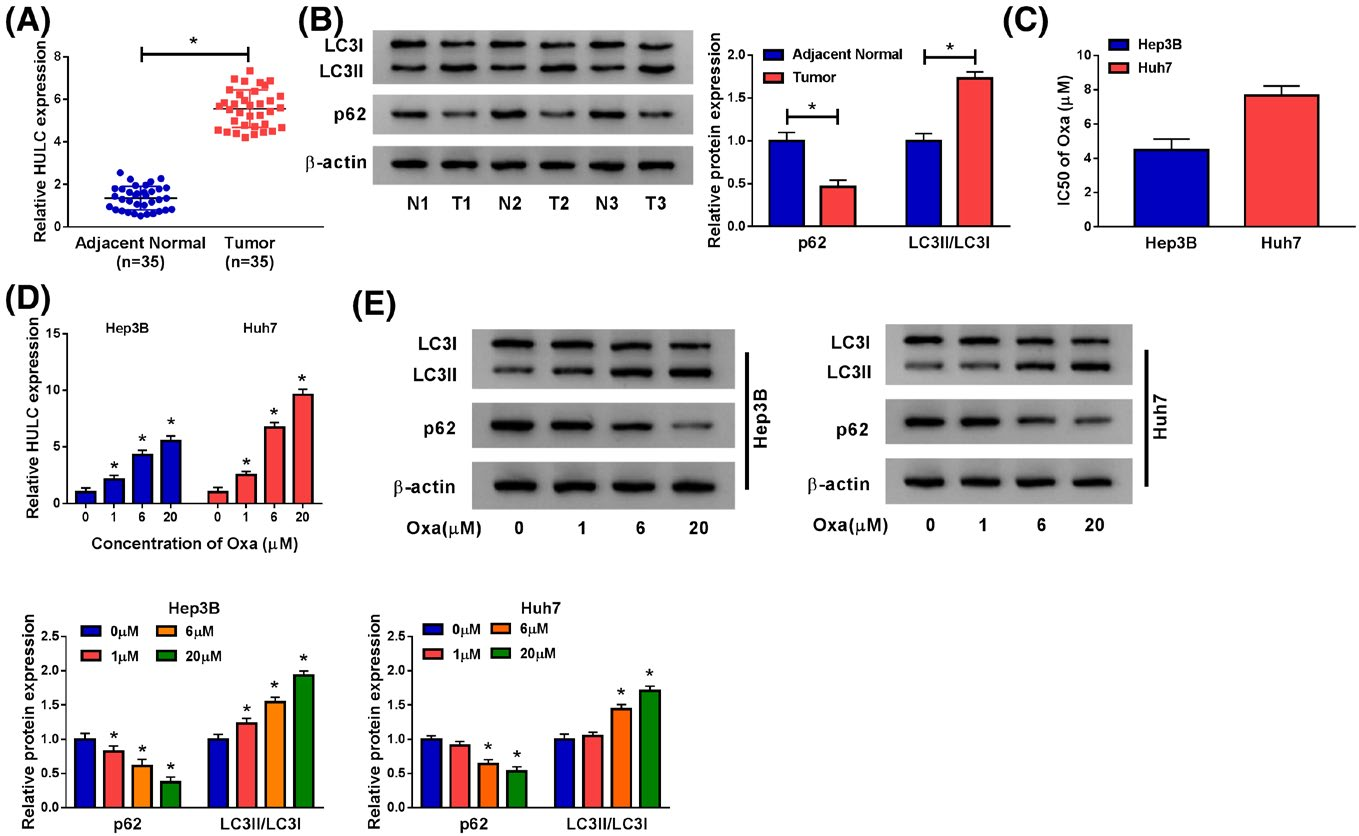
HULC was upregulated in HCC tissues and Oxa induced autophagy in HCC tissues. (A) The expression of HULC was detected by qRT‐PCR in adjacent normal tissues and HCC tissues (*t*‐test). (B) Western blot assay was used for measuring the protein levels of LC3I/II and p62 in adjacent normal tissues and HCC tissues (*t*‐test). (C) IC50 of Oxa value was tested by CCK‐8 assay in Hep3B and Huh7 cells exposed to different concentrations of Oxa (0, 0.5, 1, 2, 5, 10, and 20 μM) (*t*‐test). (D) The level of HULC was measured by qRT‐PCR in Hep3B and Huh7 cells were treated with different concentrations of Oxa (0, 1, 6, and 20 μM) (ANOVA). (E) Western blot assay was conducted to examine the protein levels of LC3I/II and p62 in Hep3B and Huh7 cells were treated with different concentrations of Oxa (ANOVA). *p < .05. ANOVA, analysis of variance; CCK‐8, Cell Counting Kit‐8; HCC, hepatocellular carcinoma; HULC, highly upregulated in liver cancer; LC3, light Chain 3; qRT‐PCR, quantitative real‐time polymerase chain reaction

To explore the potential role of HULC in HCC, we investigated HULC expression in HCC tissues and the corresponding adjacent normal tissues. The results of qRT‐PCR showed that HULC expression was obviously higher in HCC tissues than that in normal tissues (Figure 1A). Next, we explored whether autophagy was associated with HCC. Western blot was used to measure the levels of autophagy‐related proteins (LC3 and p62). LC3 is a special protein in the early stage of autophagy, and LC3I is converted to LC3II during autophagy, and p62 degradation is another marker of autophagy.^21^, ^22^ The data presented that LC3II/LC3I ratio was enhanced, suggesting that LC3I was converted to LC3II, as well as p62 expression was decreased in HCC tissues relative to normal tissues (Figure 1B). To investigate the effect of Oxa on cell viability, CCK‐8 assay was conducted in Hep3B and Huh7 cells treated with different concentrations of Oxa (0, 0.5, 1, 2, 5, 10, and 20 μM). The results presented that Oxa IC50 value was 4.48 ± 0.65 (μM) in Hep3B and 7.69 ± 0.54 (μM) in Huh7 cells (Figure 1C). Moreover, we found that HULC had a pivotal role in the sensitivity of HCC cells in response to Oxa. As presented in Figure 1D, Oxa dramatically enhanced the expression of HULC in Hep3B and Huh7 cells in a dose‐dependent manner. Furthermore, we uncovered that Oxa treatment dose‐dependently increased LC3II/LC3I ratio and decreased p62 expression in Hep3B and Huh7 cells (Figure 1E), indicating that Oxa treatment induced protective autophagy. These results suggested that HULC might act as an oncogene in HCC.

### 3.2 HULC promoted proliferation and autophagy whilst inhibited apoptosis and chemosensitivity of Oxa

**FIGURE 2:**
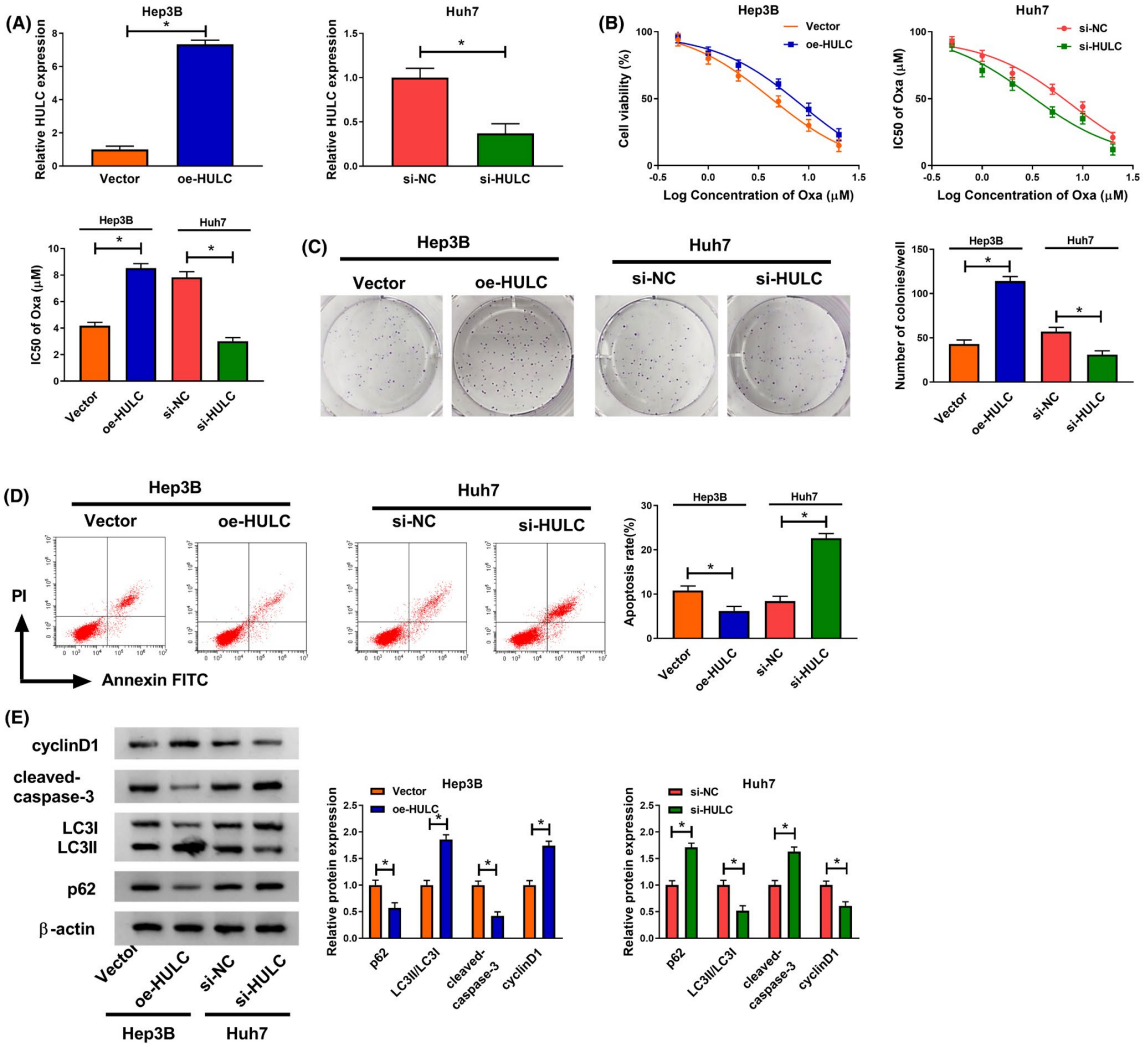
HULC promoted the progression of HCC cells and chemosensitivity of Oxa. (A) Transfection efficiency of oe‐HULC in Hep3B cells and transfection efficiency of si‐HULC in Huh7 cells were detected by qRT‐PCR (*t*‐test). (B) Cell viability and IC50 of Oxa value were measured by CCK‐8 assay in Hep3B cells transfected with Vector or oe‐HULC or Huh7 cells transfected with si‐NC or si‐HULC before treatment with different concentrations of Oxa (0, 0.5, 1, 2, 5, 10, and 20 μM) (*t*‐test). (C–E) Hep3B cells were transfected with Vector or oe‐HULC and Huh7 cells were transfected with si‐NC or si‐HULC and then these cells were treated with Oxa (6 μM). (C) Colony formation assay was utilized to determine the number of colonies (*t*‐test). (D) Cell apoptosis was determined using flow cytometry analysis (*t*‐test). (E) Western blot assay was applied to determine the protein levels of cyclinD1 cleaved‐caspase‐3, LC3I/II, and p62 (*t*‐test). *p < .05. CCK‐8, Cell Counting Kit‐8; HCC, hepatocellular carcinoma; HULC, highly upregulated in liver cancer; LC3, light Chain 3; NC, negative control; qRT‐PCR, quantitative real‐time polymerase chain reaction

First, qRT‐PCR was used to determine the transfection efficiency of oe‐HULC and si‐HULC. As displayed in Figure 2A, the expression of HULC was increased in Hep3B cells transfected with oe‐HULC, whereas its expression was decreased in Huh7 cells transfected with si‐HULC. CCK‐8 assay indicated that cell viability and Oxa IC50 value were enhanced in Hep3B cells transfected with oe‐HULC, whereas cell viability and Oxa IC50 value were reduced in Huh7 cells transfected with si‐HULC (Figure 2B). Hep3B and Huh7 cells were treated with Oxa (6 μM) for subsequent experiments. Colony formation assay indicated that overexpression of HULC increased the number of colonies in Hep3B cells, whereas knockdown of HULC presented an opposite effect in Huh7 cells (Figure 2C). Flow cytometry analysis indicated that the apoptotic rate was decreased in Hep3B cells after transfection of oe‐HULC, and interference of HULC promoted apoptosis in Huh7 cells (Figure 2D). Western blot assay indicated that upregulation of HULC increased the protein expression of cyclinD1 (regulator of cell cycle progression and cell growth) and LC3II/LC3I ratio, as well as decreased the protein levels of cleaved‐caspase‐3 (a key executor in apoptotic process) and p62 in Hep3B cells, whereas HULC silence showed opposite effects in Huh7 cells (Figure 2E). These data suggested HULC promoted the progression of HCC and suppressed the chemosensitivity of Oxa.

### 3.3 MiR‐383‐5p was a direct target of HULC

**FIGURE 3:**
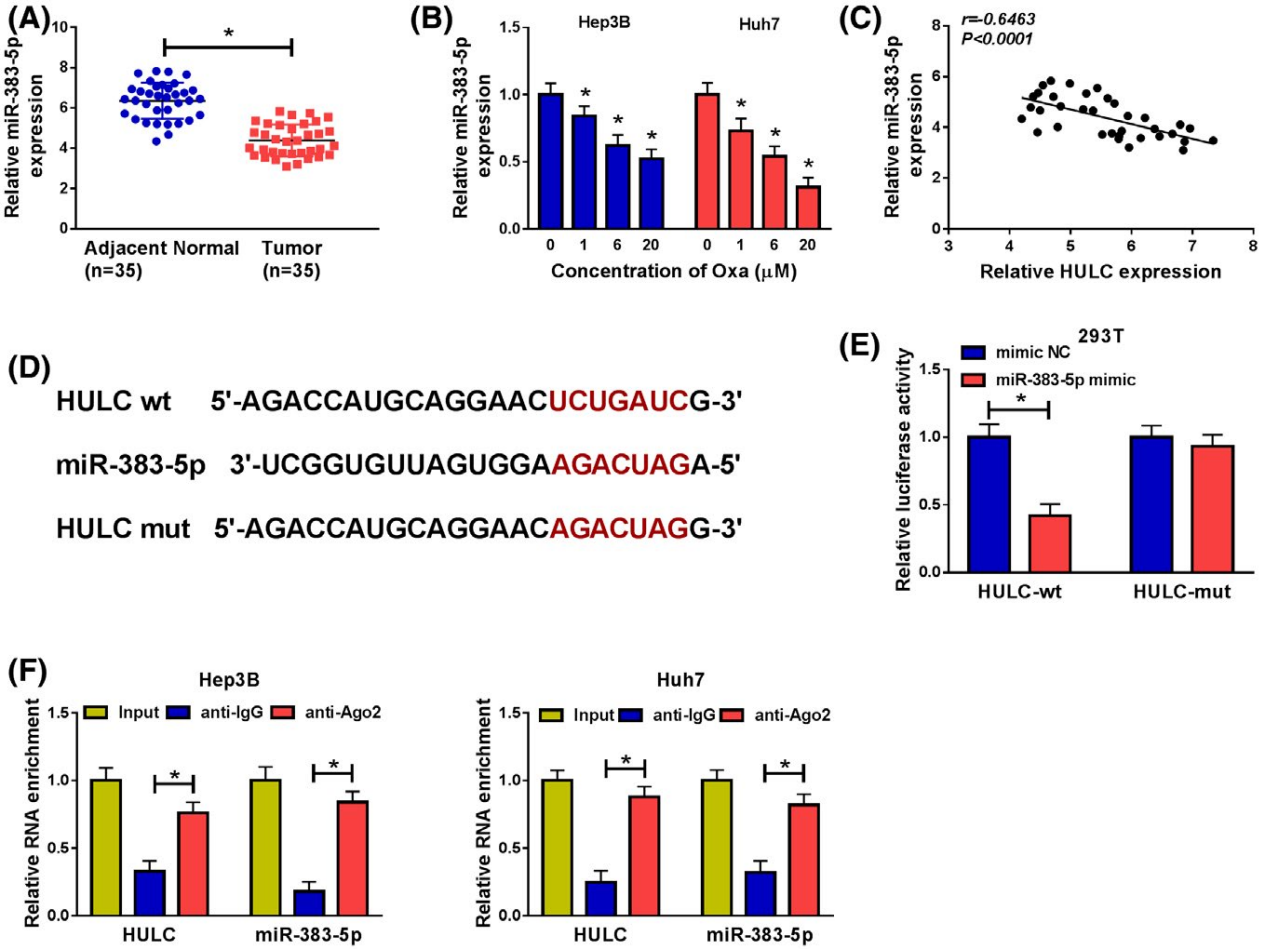
HULC directly interacted with miR‐383‐5p. (A) The abundance of miR‐383‐5p was measured by qRT‐PCR in adjacent normal tissues and HCC tissues (*t*‐test). (B) The level of miR‐383‐5p was evaluated by qRT‐PCR in Hep3B and Huh7 cells treated with different concentrations of Oxa (*t*‐test). (C) The correlation between HULC and miR‐383‐5p was analyzed in HCC tissues. (D) Predicted binding sites between HULC and miR‐383‐5p are shown. (E) The luciferase activity was measured in 293T cells co‐transfected with HULC‐wt or HULC‐mut or and miR‐383‐5p mimic or mimic NC (*t*‐test). (F) The enrichment of HULC or miR‐383‐5p was detected by RIP assay in Hep3B and Huh7 cells incubated with anti‐Ago2 or anti‐IgG (ANOVA). *p < .05. ANOVA, analysis of variance; HCC, hepatocellular carcinoma; HULC, highly upregulated in liver cancer; NC, negative control; qRT‐PCR, quantitative real‐time polymerase chain reaction

To explore the expression of miR‐383‐5p in HCC, we performed qRT‐PCR assay. As illustrated in Figure 3A, the level of miR‐383‐5p was decreased in HCC tissues in comparison with normal tissues. Moreover, Oxa treatment decreased the expression of miR‐383‐5p in a dose‐dependent manner in Hep3B and Huh7 cells (Figure 3B). In addition, the correlation between miR‐383‐5p and HULC expression was analyzed in HCC tissues. As shown in Figure 3C, miR‐383‐5p abundance was negatively correlated with HULC level in HCC tissues (*r* = −.6463, *p* < .0001). Next, we further explored the interaction between miR‐383‐5p and HULC in HCC. Bioinformatics analysis (miRcode) provided the putative binding sites of miR‐383‐5p and HULC (Figure 3D), implying the potential relationship between miR‐383‐5p and HULC. Subsequently, the prediction was validated by dual‐luciferase reporter assay and RIP assay. The data presented that the luciferase activity of HULC‐wt was significantly decreased in 293T cells transfected with miR‐383‐5p mimic, but the luciferase activity of HULC‐mut was not evidently affected after transfection with miR‐383‐5p mimic (Figure 3E). Moreover, RIP assay showed that enrichment of miR‐383‐5p and HULC was obviously enhanced in anti‐Ago2 group compared with that in anti‐IgG group (Figure 3F). Taken together, these results indicated that miR‐383‐5p could bind to HULC.

### 3.4 HULC exerted its functions by regulating miR‐383‐5p in HCC cells

**FIGURE 4:**
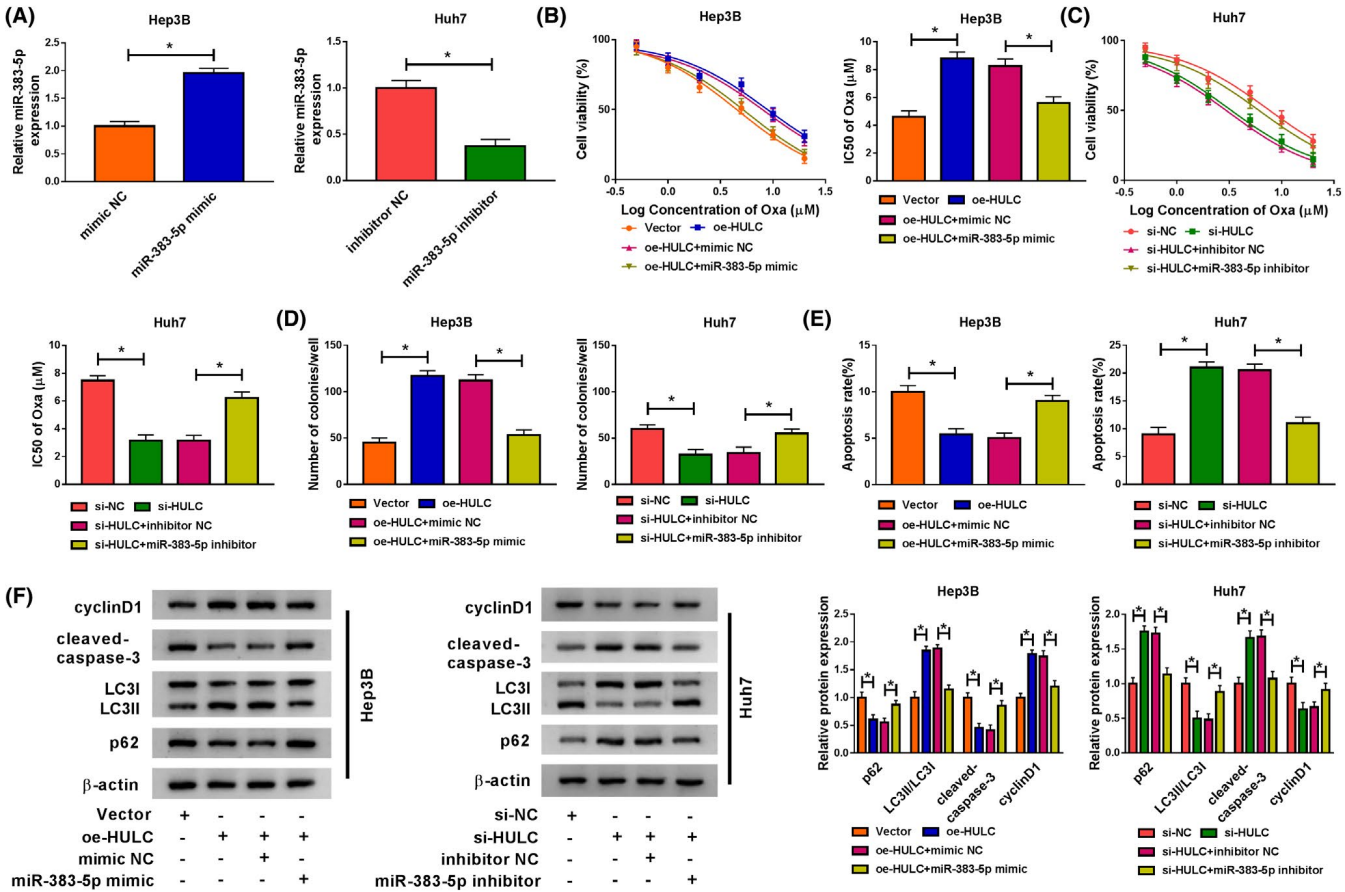
MiR‐383‐5p reversed the effects of HULC on HCC progression and chemosensitivity of Oxa. (A) Transfection efficiency of miR‐383‐5p mimic in Hep3B cells and transfection efficiency of miR‐383‐5p inhibitor in Huh7 cells were measured by qRT‐PCR (*t*‐test). (B–F) Hep3B cells were transfected with Vector, oe‐HULC, oe‐HULC + mimic NC, or oe‐HULC + miR‐383‐5p mimic and Huh7 cells were transfected with si‐NC, si‐HULC, si‐HULC + inhibitor NC, si‐HULC + miR‐383‐5p inhibitor, and then these cells were treated with different concentrations of Oxa (B,C) or 6 μM Oxa (D–F). (B,C) CCK‐8 assay was conducted to determine the cell viability and IC50 of Oxa value (ANOVA). (D) The number of colonies was examined by colony formation assay (ANOVA). (E) Cell apoptosis was determined using flow cytometry analysis (ANOVA). (F) Western blot assay was performed to measure the protein levels of cyclinD1 cleaved‐caspase‐3, LC3I/II, and p62 (ANOVA). *p < .05. ANOVA, analysis of variance; CCK‐8, Cell Counting Kit‐8; HCC, hepatocellular carcinoma; HULC, highly upregulated in liver cancer; LC3, light Chain 3; NC, negative control; qRT‐PCR, quantitative real‐time polymerase chain reaction

QRT‐PCR analysis was used to examine the transfection efficiency. The results showed that transfection of miR‐383‐5p mimic increased the expression of miR‐383‐5p in Hep3B cells, and the level of miR‐383‐5p was decreased in Huh7 cells transfected with miR‐383‐5p inhibitor (Figure 4A), suggesting that miR‐383‐5p mimic and miR‐383‐5p inhibitor were successfully transfected into Hep3B and Huh7 cells, respectively. To probe whether the effect of HULC was mediated by miR‐383‐5p, rescue experiments were performed. CCK‐8 assay indicated that overexpression of miR‐383‐5p could reverse the effect of oe‐HULC on the promotion of cell viability and Oxa IC50 value in Hep3B cells (Figure 4B), and inhibition of miR‐383‐5p abated the inhibitory effects of si‐HULC on Huh7 cell viability and Oxa IC50 value (Figure 4C). Next, Hep3B and Huh7 cells were treated with Oxa (6 μM) for subsequent analysis. Promotive effect of oe‐HULC or suppressive impact of si‐HULC on colony formation ability was abolished by upregulating miR‐383‐5p in Hep3B cells or downregulating miR‐383‐5p in Huh7 cells, respectively (Figure 4D). Moreover, overexpression of miR‐383‐5p reversed the anti‐apoptosis effect caused by upregulation of HULC, and interference of miR‐383‐5p weakened si‐HULC‐induced apoptosis (Figure 4E). In addition, the effects of HULC upregulation on the promotion of cyclinD1 expression and LC3II/LC3I ratio as well as reduction of cleaved‐caspase‐3 and p62 protein levels were abated by transfection of miR‐383‐5p mimic in Hep3B cells, and silencing miR‐383‐5p also abolished si‐HULC‐mediated opposite effects (Figure 4F). Collectively, these findings suggested that miR‐383‐5p could reverse the effects of HULC on the progression of HCC cells and chemosensitivity of Oxa.

### 3.5 VAMP2 was a downstream target of miR‐383‐5p

**FIGURE 5:**
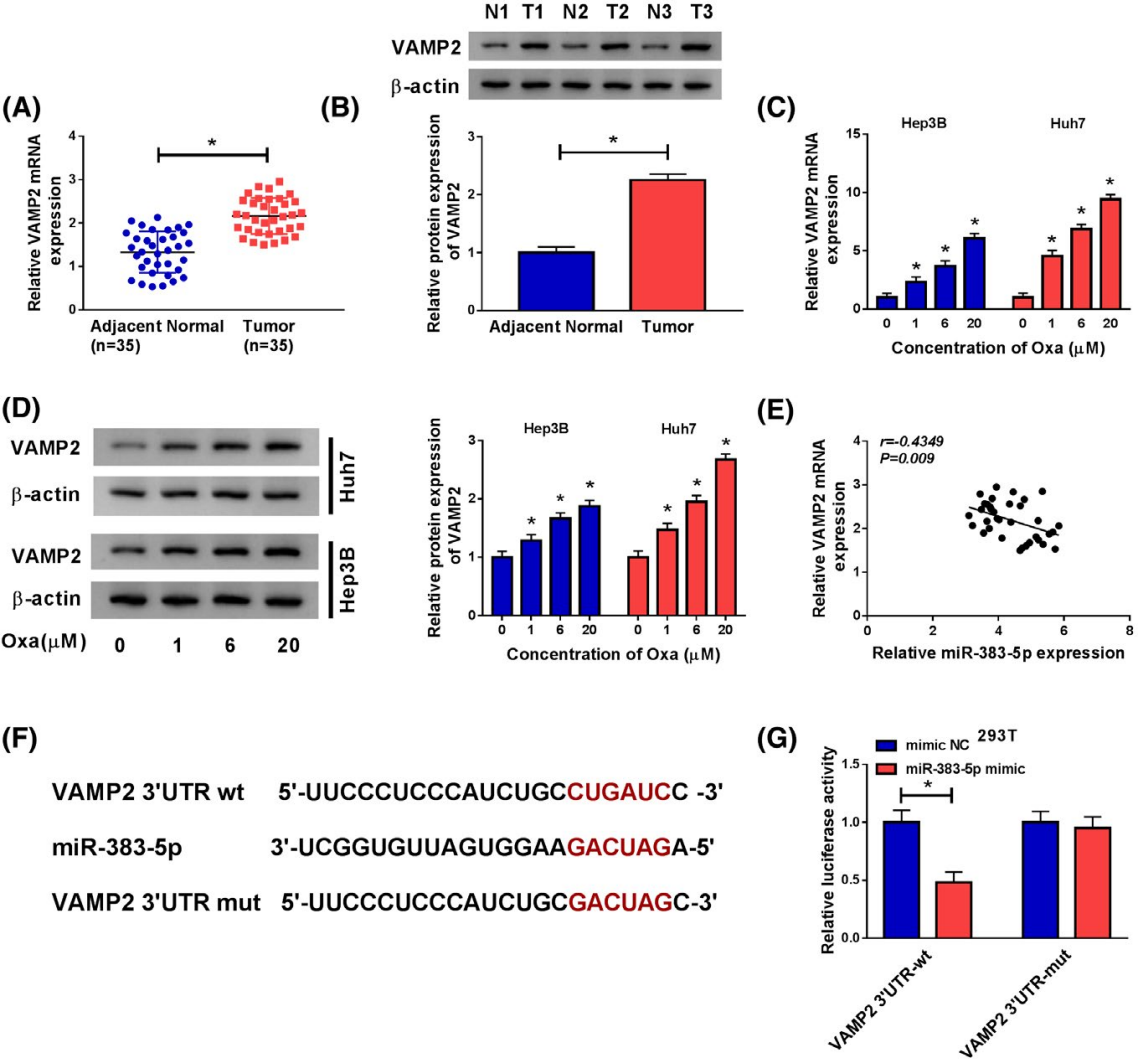
MiR‐383‐5p directly targeted VAMP2. (A,B) The mRNA expression and protein expression of VAMP2 were analyzed in adjacent normal tissues and HCC tissues by qRT‐PCR and western blot assays (*t*‐test). (C,D) The mRNA and protein levels of VAMP2 were detected in Hep3B and Huh7 cells treated with different concentrations of Oxa by qRT‐PCR and western blot analyses (ANOVA). (E) The association between miR‐383‐5p abundance and VAMP2 mRNA level was measured in HCC tissues. (F) The complementary binding sequence of miR‐383‐5p and VAMP2 was predicted by starbase v3.0. (G) Dual‐luciferase reporter assay was conducted to determine the luciferase activity in 293T cells transfected with VAMP2 3′UTR‐wt or VAMP2 3′UTR‐mut and miR‐383‐5p mimic or mimic NC (*t*‐test). *p < .05. 3′UTR, 3′‐untranslated region; ANOVA, analysis of variance; HCC, hepatocellular carcinoma; NC, negative control; qRT‐PCR, quantitative real‐time polymerase chain reaction; VAMP2, vesicle‐associated membrane protein‐2

To investigate the expression of VAMP2 in HCC tissues, VAMP2 mRNA and protein levels were determined by qRT‐PCR and western blot assays, respectively. The results displayed that mRNA and protein expression of VAMP2 were elevated in HCC tissues with respect to normal tissues (Figure 5A,B). Moreover, Oxa treatment dose‐dependently increased the mRNA and protein levels of VAMP2 in Hep3B and Huh7 cells (Figure 5C,D). Furthermore, VAMP2 mRNA level was inversely correlated with miR‐383‐5p expression in HCC tissues (Figure 5E) (*r* = −.4349, *p* = .009). Interestingly, we found that VAMP2 contained putative binding sites of miR‐383‐5p in the 3′UTR (Figure 5F). To verify whether VAMP2 was a direct target of miR‐383‐5p, the dual‐luciferase reporter assay was carried out. Results indicated that overexpression of miR‐383‐5p greatly reduced the luciferase activity of VAMP2 3′UTR‐wt in 293T cells, whereas miR‐383‐5p upregulation had no impact on the luciferase activity of VAMP2 3′UTR‐mut (Figure 5G). In a word, these data indicated that miR‐383‐5p directly targeted VAMP2.

### 3.6 MiR‐383‐5p exerted its effect by modulating YWHA in HCC cells

**FIGURE 6:**
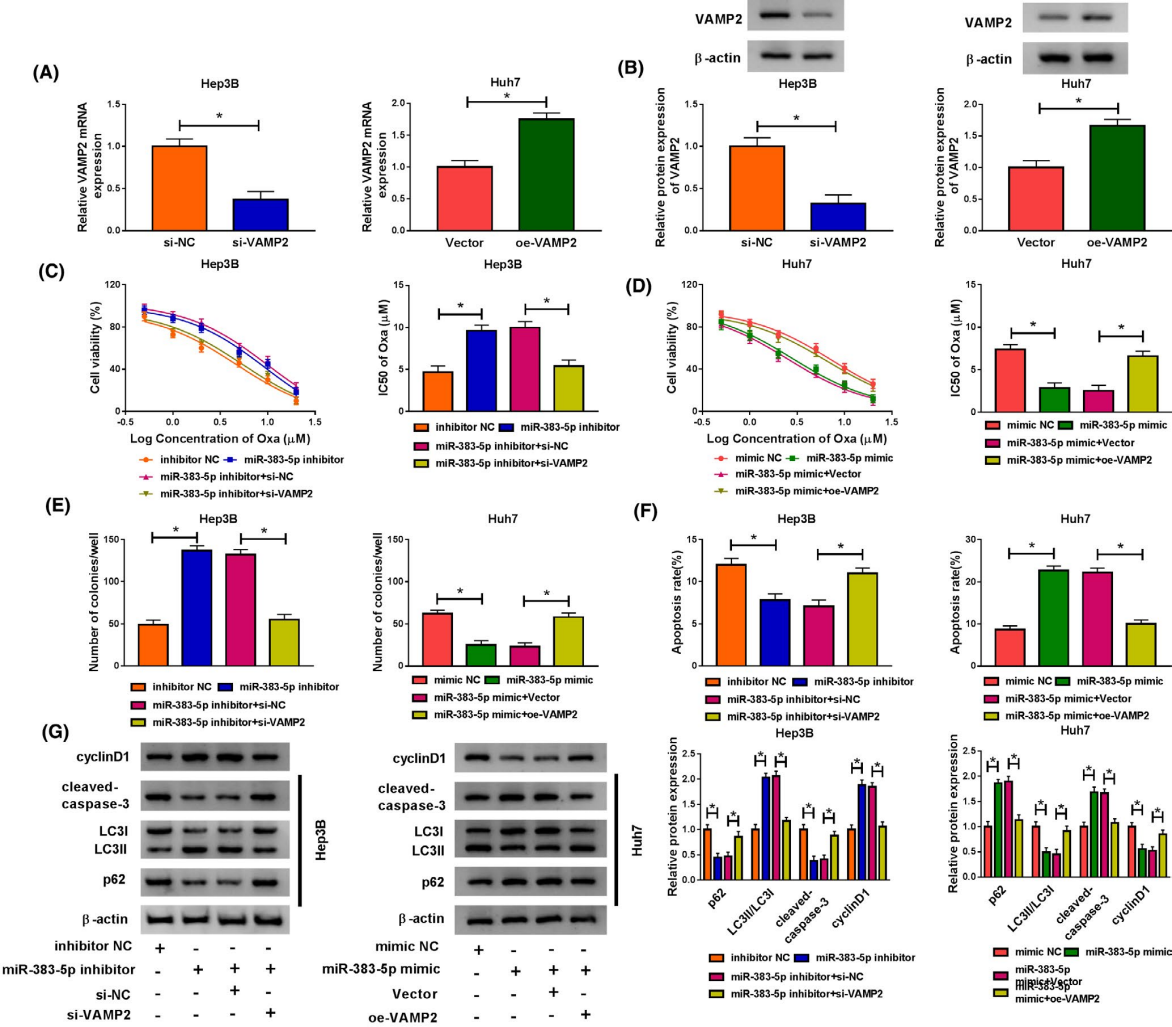
VAMP2 abated the functions of miR‐383‐5p on HCC progression and chemosensitivity of Oxa. (A,B) Transfection efficiency of si‐VAMP2 in Hep3B cells and transfection efficiency of oe‐VAMP2 in Huh7 cells were determined by qRT‐PCR and western blot assays (*t*‐test). (C–G) Hep3B cells were transfected with inhibitor NC, miR‐383‐5p inhibitor, miR‐383‐5p inhibitor + si‐NC, or miR‐383‐5p inhibitor + si‐VAMP2 and Huh7 cells were transfected with mimic NC, miR‐383‐5p mimic, miR‐383‐5p mimic + Vector, or miR‐383‐5p mimic + oe‐VAMP2, and then these cells were treated with different concentrations of Oxa (C,D) or 6 μM Oxa (E–G). (C,D) CCK‐8 assay was utilized to assess the cell viability and IC50 of Oxa value (ANOVA). (E) Colony formation ability was evaluated using a colony formation assay (ANOVA). (F) Cell apoptosis was analyzed using flow cytometry analysis (ANOVA). (G) Western blot assay was carried out to measure the protein levels of cyclinD1 cleaved‐caspase‐3, LC3I/II, and p62 (ANOVA). *p < .05. ANOVA, analysis of variance; CCK‐8, Cell Counting Kit‐8; HCC, hepatocellular carcinoma; LC3, light Chain 3; NC, negative control; qRT‐PCR, quantitative real‐time polymerase chain reaction; VAMP2, vesicle‐associated membrane protein‐2

Next, we further validated the association between VAMP2 and miR‐383‐5p in HCC cells. The results from qRT‐PCR and western blot indicated that VAMP2 mRNA and protein levels were decreased in Hep3B cells transfected with si‐VAMP2 and VAMP2 mRNA and protein levels were increased in Huh7 cells transfected with oe‐VAMP2 (Figure 6A,B), suggesting that si‐VAMP2 and oe‐VAMP2 were successfully transfected into Hep3B cells and Huh7 cells, respectively. CCK‐8 assay demonstrated that interference of miR‐383‐5p increased cell viability and Oxa IC50 value in Hep3B cells or overexpression of miR‐383‐5p decreased cell viability and Oxa IC50 value in Huh7 cells, whereas these effects were abated by downregulating VAMP2 or upregulating VAMP2 (Figure 6C,D). Subsequently, Hep3B and Huh7 cells were exposed to Oxa (6 μM) for subsequent assays. Downregulation of miR‐383‐5p elevated the number of colonies in Hep3B cells or restoration of miR‐383‐5p reduced the number of colonies in Huh7 cells, which was abrogated by knockdown of VAMP2 or overexpression of VAMP2 (Figure 6E). Furthermore, silencing VAMP2 overturned the anti‐apoptosis effect caused by miR‐383‐5p inhibitor in Hep3B cells, and overexpression of VAMP2 abated the promotive effect of miR‐383‐5p mimic on apoptosis in Huh7 cells (Figure 6F). Besides, the effects of miR‐383‐5p interference on increase in cyclinD1 expression and LC3II/LC3I ratio and decrease in cleaved‐caspase‐3 and p62 expression in Hep3B cells or miR‐383‐5p‐mediated opposite effects were abolished through knockdown of VAMP2 or overexpression of VAMP2 (Figure 6G). These data illustrated that VAMP2 could reverse the functions of miR‐383‐5p on the progression of HCC cells and chemosensitivity of Oxa.

### 3.7 VAMP2 was regulated by HULC and miR‐383‐5p in HCC cells

**FIGURE 7:**
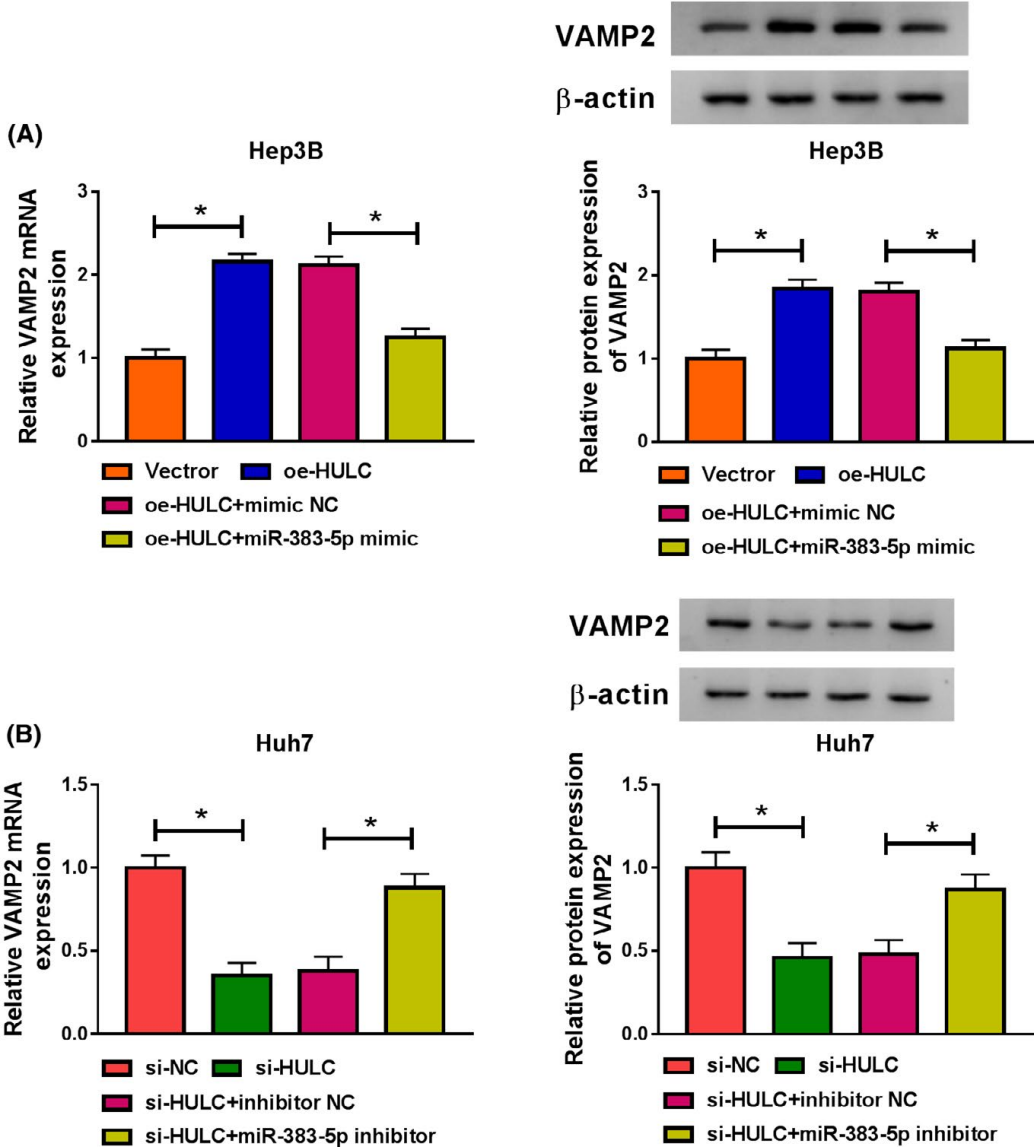
HULC positively regulated VAMP2 expression by sponging miR‐383‐5p in HCC cells. (A) The mRNA expression and protein expression of VAMP2 were determined by qRT‐PCR and western blot analyses, respectively, in Hep3B cells transfected with Vector, oe‐HULC, oe‐HULC + mimic NC, or oe‐HULC + miR‐383‐5p mimic (ANOVA). (B) QRT‐PCR and western blot analyses were conducted to analyze the mRNA level and protein level of VAMP2 in Huh7 cells transfected with si‐NC, si‐HULC, si‐HULC + inhibitor NC, or si‐HULC + miR‐383‐5p inhibitor (ANOVA). *p < .05. ANOVA, analysis of variance; HCC, hepatocellular carcinoma; HULC, highly upregulated in liver cancer; NC, negative control; qRT‐PCR, quantitative real‐time polymerase chain reaction; VAMP2, vesicle‐associated membrane protein‐2

Next, we tried to investigate whether HULC served as a ceRNA of miR‐383‐5p to modulate VAMP2 expression. The results from qRT‐PCR and western blot showed that overexpression of HULC increased the mRNA and protein levels of VAMP2 in Hep3B cells, which were weakened by upregulating miR‐383‐5p (Figure 7A). Moreover, the mRNA and protein levels of VAMP2 were reduced in Huh7 cells after transfection with si‐HULC, whereas the effect was reversed by downregulating miR‐383‐5p (Figure 7B). These above findings indicated that HULC regulated VAMP2 expression by sponging miR‐383‐5p in HCCcells.

### 3.8 Overexpression of HULC accelerated tumor growth by regulating miR‐383‐5p and VAMP2 in vivo

**FIGURE 8:**
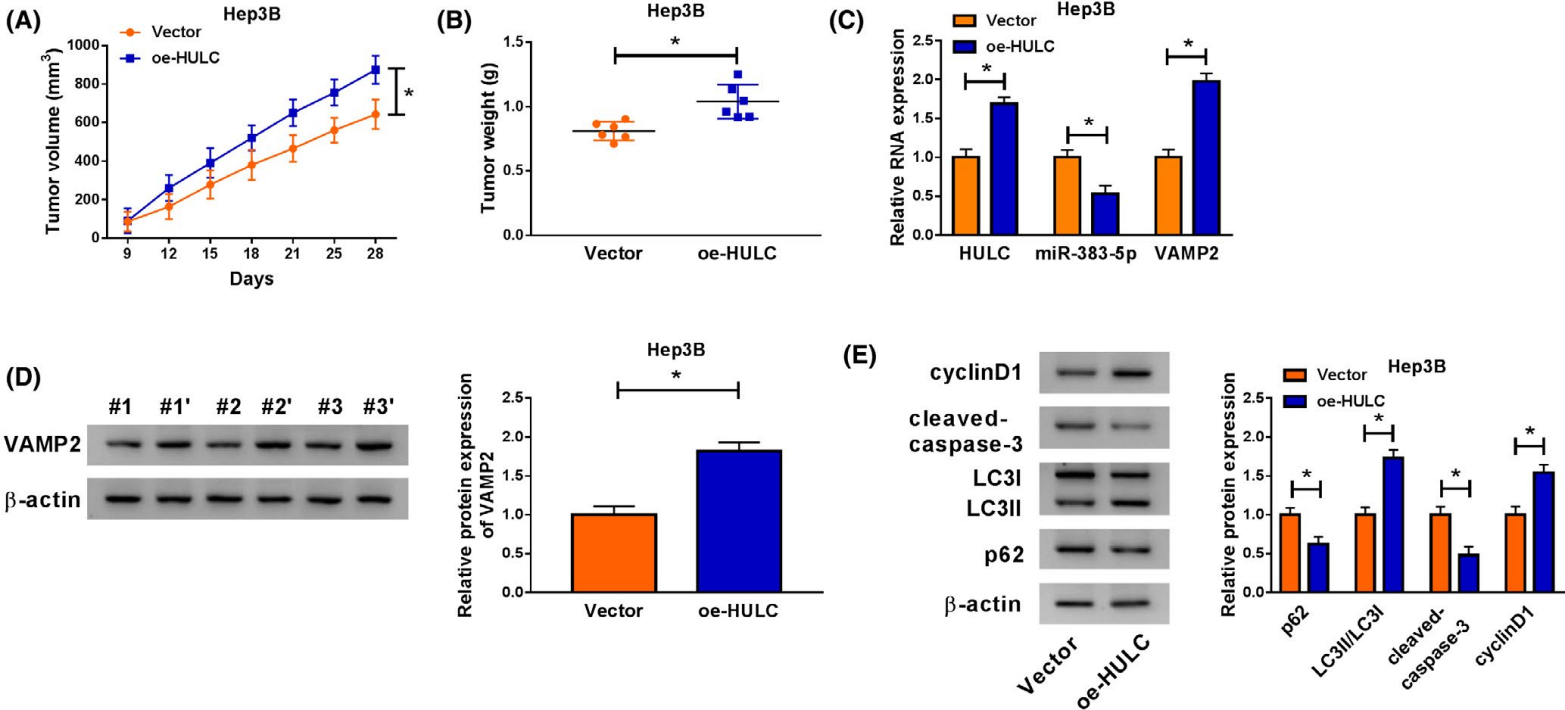
Upregulation of HULC promoted tumor growth and inhibited chemosensitivity of Oxa by regulating miR‐383‐5p and VAMP2. Hep3B cells transfected with Vector or oe‐HULC were inoculated subcutaneously into the nude mice. The mice were treated with Oxa (5 mg/kg) twice a week after injection for 3 days. (A,B) Tumor volume and weight were measured (ANOVA). (C) The expression levels of HULC, miR‐383‐5p, and VAMP2 were examined by qRT‐PCR in resected tumor tissues (*t*‐test). (D) Western blot assay was applied to detect the protein abundance of VAMP2 in resected tumor masses (*t*‐test). (E) The protein levels of cyclinD1, cleaved‐caspase‐3, LC3I/II, and p62 were analyzed in resected tumor tissues using western blot analysis (*t*‐test). *p < .05. ANOVA, analysis of variance; HULC, highly upregulated in liver cancer; LC3, light Chain 3; qRT‐PCR, quantitative real‐time polymerase chain reaction; VAMP2, vesicle‐associated membrane protein‐2

To explore the impact of HULC on tumor growth and chemosensitivity in vivo, we established a xenograft model in which the Hep3B cells stably transfected with Vector or oe‐HULC were subcutaneously injected into BALB/c nude mice and treated with Oxa (5 mg/kg) twice a week. In agreement with in vitro results, overexpression of HULC increased tumor volume and weight in the xenograft model compared with the control group (Figure 8A,B). Moreover, upregulation of HULC increased the expression of HULC and VAMP2 as well as decreased the abundance of miR‐383‐5p in resected tumor tissues (Figure 8C). Meanwhile, enforced expression of HULC also enhanced the protein level of VAMP2 in resected tumor tissues (Figure 8D). Additionally, overexpression of HULC enhanced the protein expression of cyclinD1 and LC3II/LC3I ratio and decreased the protein levels of cleaved‐caspase‐3 and p62 in resected tumor tissues (Figure 8E). Therefore, these results revealed that HULC overexpression could promote tumor growth and inhibit chemosensitivity of Oxa by downregulating miR‐383‐5p and upregulating VAMP2.

## 4 DISCUSSION

Hepatocellular carcinoma is an aggressive malignant tumor with a high recurrence rate.^23^ Although Oxa‐based chemotherapy is especially effective in a variety of cancers, chemoresistance development has become a major obstacle in cancer treatment.^24^, ^25^ Numerous lncRNAs have been suggested to participate in modulating tumor processes and chemosensitivity.^26^, ^27^ In this research, we focused on the biological role and underlying mechanism of HULC in HCC progression and chemosensitivity of Oxa.

Mounting evidence indicated that HULC played pivotal roles in HCC development. For instance, Xiong et al. demonstrated that HULC promoted HCC cell growth by stabilizing COX‐2 protein.^28^ Xin et al. revealed that HULC could accelerate the malignant progression of HCC through suppressing PTEN via autophagy cooperation to miR‐15a.^29^ Protective autophagy has been shown to be a possible mechanism for chemoresistance in various types of cancer cells, and the promotion of apoptosis is often beneficial to improve the sensitivity of chemotherapy drugs.^19^, ^30^, ^31^ Notably, Xiong and his colleagues found that HULC‐induced protective autophagy weakened the chemosensitivity of antitumor reagents in HCC cells.^13^ In line with these findings, our results also proved that HULC was overexpressed in HCC tissues and its overexpression decreased the chemosensitivity of Oxa by triggering autophagy and inhibiting cell apoptosis. Meanwhile, HULC knockdown repressed cell growth and autophagy as well as accelerated apoptosis in HCC cells treated with Oxa, suggesting that HULC knockdown increased the chemosensitivity of Oxa.

Previous reports proved that miR‐383‐5p acted as an anti‐oncogene in diverse tumors, such as ovarian cancer,^32^ breast cancer,^33^ and gastric cancer.^34^ Besides, Chen et al. stated that miR‐383 abundance was declined in HCC tissues and cells and its overexpression limited HCC cell proliferation by targeting APRIL.^20^ Wang et al. pointed out that miR‐383 limited HCC cell growth and facilitated cell apoptosis via regulating IL‐17 and STAT3 signaling pathway.^35^ Consistent with these previous findings, we also observed that miR‐383‐5p abundance was declined in HCC tissues. Besides, miR‐383‐5p expression was inversely associated with HULC level in HCC tissues. Interestingly, a large number of evidence has suggested that lncRNAs may function as ceRNAs or miRNA sponges to affect miRNAs, resulting in a change in the expression of miRNA target gene.^36^ To probe whether HULC served as a molecular sponge of miR‐383‐5p, we predicted their targeting relationship by miRcode. As expected, miR‐383‐5p was identified as a direct target of HULC. Furthermore, we uncovered that miR‐383‐5p could abolish the functions of HULC on cell growth, apoptosis, and autophagy as well as chemosensitivity of Oxa. These results indicated that HULC exerted its function by sponging miR‐383‐5p in HCC cells.

To date, VAMP2 was reported to be aberrantly expressed in some tumors and played important regulative roles.^37^, ^38^ More importantly, Wang et al. proved that VAMP2 level was upregulated in HCC tissues and its upregulation reversed the anti‐cancer role of miR‐493‐5p in liver cancer cells.^39^ Here, we also uncovered that VAMP2 mRNA and protein expression were enhanced in HCC tissue samples, and its mRNA level was inversely associated with miR‐383‐5p abundance in HCC tissues. Using the online software starbase v3.0, it was predicted that VAMP2 might bind with miR‐383‐5p. Next, our results verified that VAMP2 could interact with miR‐383‐5p. Moreover, rescue experiments indicated that VAMP2 could abate the impact of miR‐383‐5p on the progression of HCC cells and chemosensitivity of Oxa in HCC cells. Additionally, HULC positively modulated VAMP2 expression via sponging miR‐383‐5p. In vivo experiments implied that HULC overexpression promoted tumor growth and inhibited the chemosensitivity of Oxa through downregulating miR‐383‐5p and upregulating VAMP2. However, HULC has a small effect on tumor growth, and this effect does not necessarily translate into meaningful clinical benefits. Therefore, further research on the role of HULC in the clinic is needed in the future. Taken together, our findings suggested that HULC regulated the progression of HCC and chemosensitivity of Oxa via modulating miR‐383‐5p/VAMP2 axis. Although lncRNAs have great potential in the diagnosis and prognosis of HCC, when lncRNAs are used as a potential drug candidate or therapeutic target for HCC treatment, we also face a series of challenges, such as lncRNAs are difficult to pass through biofilms and lncRNAs can be quickly eliminated by the liver and kidneys. Presently, there are two main methods to solve these problems: One is to modify nucleic acid molecules to stabilize and avoid recognition by the immune system, the other is to use drug delivery systems, such as nanoparticle vector technology, protamine vector technology, adeno‐associated viral vector technology. Nevertheless, these delivery systems still have defects such as immune system rejection and reduced protein expression efficiency, and future research should focus on developing new methods to overcome these shortcomings.

In conclusion, we discovered that HULC and VAMP2 were overexpressed whilst miR‐383‐5p was lowly expressed in HCC tissues. Functionally, HULC overexpression promoted the progression of HCC and attenuated the chemosensitivity of Oxa by regulating miR‐383‐5p/VAMP2 axis. Hence, our study might contribute to a better understanding of the regulatory mechanism of HCC progression and chemosensitivity of Oxa, offering a promising lncRNA‐targeted therapy for HCC.

## DISCLOSURE

The authors declare that they have no competing interests.

## ETHICS APPROVAL AND CONSENT TO PARTICIPATE

This study was approved by the ethical review committee of First Affiliated Hospital of Xi'an Jiaotong University. Written informed consent was obtained from all enrolled patients.

## CONSENT FOR PUBLICATION

Patients agree to participate in this work.

## Data Availability

The analyzed datasets generated during this study are available from the corresponding author on reasonable request.
